# A genetic code alteration generates a proteome of high diversity in the human pathogen *Candida albicans*

**DOI:** 10.1186/gb-2007-8-10-r206

**Published:** 2007-10-04

**Authors:** Ana C Gomes, Isabel Miranda, Raquel M Silva, Gabriela R Moura, Benjamin Thomas, Alexandre Akoulitchev, Manuel AS Santos

**Affiliations:** 1 CESAM & Department of Biology, University of Aveiro, 3810-193 Aveiro, Portugal; 2 Central Proteomics Facility, Sir William Dunn School of Pathology, University of Oxford, South Parks Road, Oxford OX1 3RE, UK

## Abstract

**Background:**

Genetic code alterations have been reported in mitochondrial, prokaryotic, and eukaryotic cytoplasmic translation systems, but their evolution and how organisms cope and survive such dramatic genetic events are not understood.

**Results:**

Here we used an unusual decoding of leucine CUG codons as serine in the main human fungal pathogen *Candida albicans *to elucidate the global impact of genetic code alterations on the proteome. We show that *C. albicans *decodes CUG codons ambiguously and tolerates partial reversion of their identity from serine back to leucine on a genome-wide scale.

**Conclusion:**

Such codon ambiguity expands the proteome of this human pathogen exponentially and is used to generate important phenotypic diversity. This study highlights novel features of *C. albicans *biology and unanticipated roles for codon ambiguity in the evolution of the genetic code.

## Abstract 2

An unusual decoding of leucine CUG codons as serine in *Candida albicans *revealed unanticipated codon ambiguity, which expands the proteome of this human pathogen exponentially.

## Background

Since the elucidation of the genetic code in the 1960s, 24 alterations in codon identity have been recorded in prokaryotic and eukaryotic translation systems. These alterations involve redefinition of identity of both sense and nonsense codons and codon unassignment (codons vanished from genomes) [1]. Furthermore, artificial expansion of the genetic code to incorporate non-natural amino acids [2-4] and natural incorporation of selenocysteine (Sec; 21st amino acid) and pyrrolysine (22nd amino acid) have also been reported [5,6]. Sec is incorporated in both prokaryotic and eukaryotic selenoproteins through reprogramming of UGA stop codons by novel translation elongation factors (selenoprotein translation factor B prokaryotes, elongation factor [EF]-Sec, and selenium-binding protein 2 eukaryotes), a new tRNA (tRNA^Sec^), and a Sec mRNA insertion element [7]. L-pyrrolysine insertion occurs in the archeon *Methanosarcina barkeri *through reprogramming of the UAG stop codon by a pyrrolysine insertion sequence in the methylamine methyltransferase mRNA [8]. The flexibility of the genetic code is further exemplified by the absence of glutamine and asparagine aminoacyl-tRNA synthetases in several mitochondria and archaeal and bacterial species. In those particular cases, aminoacylation of tRNA^Gln ^and tRNA^Asn ^is accomplished by an ATP-dependent transamidation reaction on mis-charged Glu-tRNA^Gln ^and Asp-tRNA^Asn ^[9-11]. *Methanococcus jannaschii*, *Methanopyrus kandleri*, and *Methanothermobacter thermoautotrophicus *all lack canonical cysteinyl-tRNA synthetases and charge tRNA^Cys ^with the intermediate substrate O-phosphoseryl (Sep), using the enzyme Sep-tRNA synthetase. Sep-tRNA^Cys ^is then converted to Cys-tRNA^Cys ^by Sep-tRNA:Cys-tRNA synthetase [12].

The unusual decoding properties described above reflect evolutionary steps in the development of the genetic code. They support the co-evolutionary theory of organization of the primordial genetic code [13] and demonstrate that most of the alterations and expansions are mediated by structural changes in the protein synthesis machinery, in particular in tRNAs, aminoacyl-tRNA synthetases, EFs and termination factors [14]. However, these data *per se *do not provide insight into the evolutionary forces that drive codon identity redefinition, and neither do they help in evaluating the impact of genetic code alterations on proteome and genome stability, gene expression, adaptation, and ultimately evolution of new phenotypes.

In order to shed new light on the above questions, we chose the human pathogen *Candida albicans *as a well studied model system [15-18]. *C. albicans *and other *Candida *spp. have a unique genetic code because of the change in the identity of the leucine CUG codon to serine, which evolved through an ambiguous codon decoding mechanism that affected approximately 30,000 CUG codons in more than 50% of the genes [19]. Because serine is polar and leucine hydrophobic, the change in identity of CUG codons across all of the open reading frames (ORFeome) must have caused major proteome disruption. This raises an important question of how the *Candida *ancestor managed to survive such a dramatic genetic event. Here, we deployed direct protein mass spectrometry analysis to shed new light on this important biologic issue. We show that the CUG codon is decoded as both serine and leucine *in vivo *and that *C. albicans *tolerates up to 28.1% of leucine mis-incorporation at CUG positions, which represents a 28,000-fold increase in decoding error. This increased dramatically the number of different proteins encoded by the 6,438 *C. albicans *genes and resulted in extensive and unanticipated phenotypic variability. The data provide new insight into the evolution of the genetic code and *C. albicans *biology, and demonstrate that alterations in genetic code are dynamic molecular processes of unexpected relevance to phenotypic diversity.

## Results

### Identity of the *C. albicans *CUG codon *in vivo*

The genetic code alteration in *Candida *is the only known case of a sense-to-sense codon identity redefinition in eukaryotes. The other cases deal with redefinition of stop codons, for instance UAR to glutamine in various ciliates and green algae, UGA to cysteine in *Euplotes *spp., and UAG to glutamate in various peritrich species [1].

**Figure 1:**
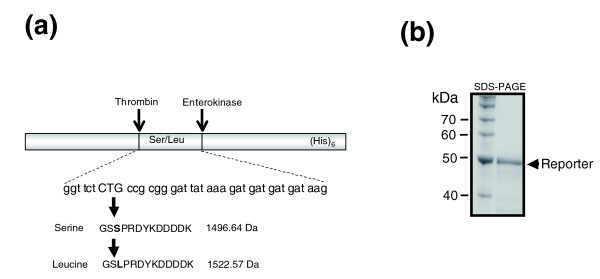
Reporter system to quantify CUG ambiguity in *Candida albicans*. **(a) **A recombinant gene, constructed by modifying the *CaPGK *gene, was used to monitor CUG ambiguity *in vivo *in *C. albicans *CAI-4 Cells. Thrombin and enterokinase sites, flanking a CUG reporter cassette, were introduced in the *CaPGK *in conjunction with a flag-tag epitope and a poly(his)_6_-tag. **(b) **The recombinant protein was expressed and purified to near homogeneity by nickel-agarose affinity chromatography. For high-pressure liquid chromatography-mass spectroscopy analysis, this protein was in-gel digested for 36 hours in presence of 3.0 × 10^-4 ^U/μl of enterokinase and 3.0 × 10^-5 ^U/μl of thrombin (Novagen).

**Figure 2:**
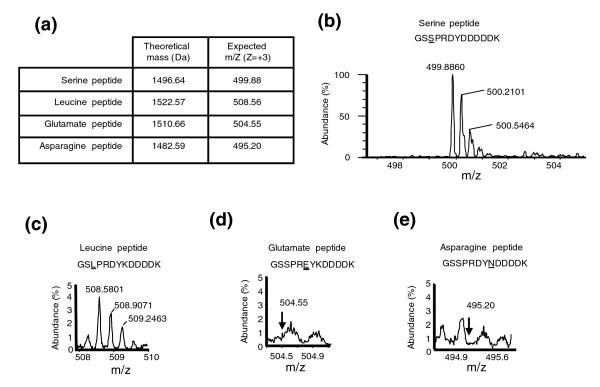
Mis-translation due to near-cognate decoding. The typical mRNA translation error *in vivo *in yeast is in the order of 10^-5^, but some codons are more prone to mis-translation than others by near-cognate tRNAs. In order to ensure that leucine mis-incorporation could be detected above background noise, the mass spectra were screened for the presence of peptides resulting from near-cognate decoding. **(a) **Table showing the theoretical mass and the expected m/Z peaks of the peptides that were screened in the mass spectroscopy experiments. The serine peptide was the product of correct translation of the recombinant gene used in the study, and it was the most abundant. The leucine peptide corresponded to a peptide synthesized by ambiguous decoding of the CUG codon by the *C. albicans *tRNA_CAG_^Ser^. The glutamate peptide was the product of decoding of the aspartate-GAU codon as glutamate by the near-cognate tRNA that decodes the glutamate GAA and GAG codons. Likewise, the lysine-AAA and AAG codons could be decoded by the near-cognate tRNAs that decode the asparagines AAU and AAC codons. **(b) **Mass spectrum of the serine peptide. **(c) **Mass spectrum of the leucine peptide. **(d) **Mass spectrum showing the region where the peak corresponding to the peptide containing glutamate at the aspartate position was expected (arrow). **(e) **Mass spectrum showing the region where the peak corresponding to the peptide containing asparagines in the position of the lysine-AAA codons was expected (arrow).

In *Candida*, the alteration in identity of the CUG codon evolved over 272 ± 25 million years through an ambiguous codon decoding mechanism [17,19]. It arose from competition of a mutant tRNA_CAG_^Ser ^with wild-type tRNA_CAG_^Leu ^and from leucine mischarging of the former tRNA [19-21]. Because the novel *C. albicans *tRNA_CAG_^Ser ^has identity elements for both seryl-tRNA synthetases and leucyl-tRNA synthetases (LeuRSs) and can still be mischarged *in vitro *with leucine [21], we investigated whether CUG codons could remain ambiguous *in vivo*. For this purpose, a reporter protein for monitoring ambiguous CUG decoding, containing an amino-terminal CUG cassette, was constructed based on the *C. albicans PGK *(phospho-glycero kinase) protein (Figure 1a). The protein was then expressed in *C. albicans *CAI-4 cells using a *C. albicans *shuttle vector (pUA63; Additional data file 1 [Figure S1A]), purified to near homogeneity (Figure 1a), and in-gel digested with enterokinase and thrombin. The resulting peptides were identified and quantified using high-pressure liquid chromatography (HPLC) and tandem mass spectrometry (Figure 2).

In order to determine whether the HPLC-mass spectrometry methodology used was adequate to quantify leucine mis-incorporation at the CUG codon, synthetic peptides of identical amino acid sequence were used (see Materials and methods, below). Furthermore, amino acid mis-incorporation at near-cognate codons was monitored to ensure that leucine mis-incorporation at the CUG position could be detected above background noise. Near-cognate misreading is the most frequent mistranslation error because it involves misreading at the wobble position by near cognate tRNAs [22]. This error has been monitored in yeast *in vivo *and is in the order of 0.001% [23]. Because the aspartate GAU and lysine AAA codons encoded by the reporter peptide (Figure 1a) could be misread by near-cognate tRNA^Glu ^and tRNA^Asn^, respectively, the mass on these aberrant peptides containing glutamate at the aspartate-GAU position or asparagine at the lysine-AAA position was determined (Figure 2a). The peptides resulting from correct serine incorporation and leucine mis-incorporation at the CUG position were clearly visible in the mass spectrum (Figure 2b,c), whereas the peptides containing serine at the CUG position plus glutamate at the aspartate-GAU position or serine at CUG plus asparagine at the lysine-AAA position were not detected (Figure 2d,e). This confirmed that our methodology was robust for accurate quantification of mistranslation of the *C. albicans *serine CUG codon as leucine.

**Figure 3:**
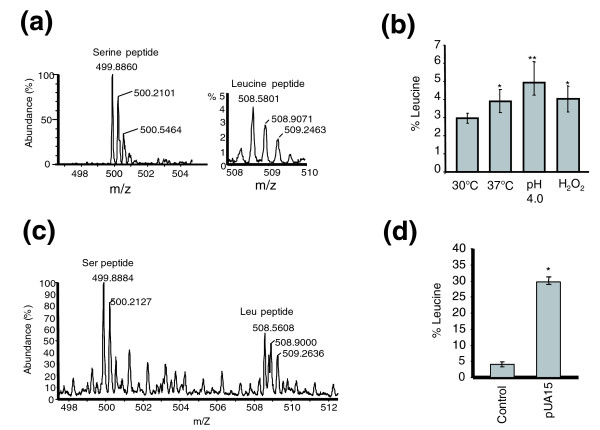
CUG ambiguity *in vivo *in *Candida albicans *in different environmental conditions. Quantification of CUG ambiguity *in vivo *was carried out using a reporter protein that contained a CUG codon cassette and a poly(His)_6 _tag. **(a,b) **Leucine mis-incorporation at the CUG position was determined in white cells at 30°C, 37°C, in pH 4.0, in 1.5 mmol/l hydrogen peroxide (H_2_O_2_), and ranged from 2.96 ± 0.49%, 3.9 ± 0.64%, 4.95 ± 1.14% to 4.03 ± 0.71%, respectively. *C. albicans *white cells were used because opaque cells are very rare and under normal growth conditions only white cells are found in culture. *P *values were determined using the Scheffe test and are as follows: *P = 0.048 and **P = 0.0017. **(c,d) **Mass spectrum of the reporter protein purified from *C. albicans *cells expressing the *Saccharomyces cerevisiae *tRNA_CAG_^Leu^, showing that 28.1% ± 1.17 of the peptides incorporated leucine and 71.9% ± 1.17 incorporated serine at the CUG codon position. *P* value is as follows; *P ≈ 0.

The levels of leucine mis-incorporation at the CUG codons were then quantified and were 2.96% in *C. albicans *white cells grown at 30°C, 3.9% at 37°C, 4.03% in presence of hydrogen peroxide (H_2_O_2_), and 4.95% at pH 4.0 (Figure 3a,b). These values represent between 2,960-fold and 4,950-fold increases in mistranslation (10^-5 ^typical error [23]) and imply that the tRNA_CAG_^Ser ^is charged *in vivo *with both serine and leucine and that the mischarged leu-tRNA_CAG_^Ser ^is neither edited by the LeuRS nor discriminated by translation elongation factor 1A.

The unexpected CUG mistranslation in wild-type cells prompted us to investigate whether the identity of the CUG codon could be reverted to leucine or whether CUG ambiguity could be tolerated at higher levels. For this, a *Saccharomyces cerevisiae *gene encoding a mutant tRNA_CAG_^Leu^, which decodes CUG codons as leucine by standard Watson-Crick base pairing, was inserted into plasmid pUA63, which already contained the CUG-reporter protein gene, producing plasmid pUA65 (Additional data file 1 [Figure S1B]). The pUA65 plasmid was then transformed into *C. albicans *CAI-4 cells. Because the recombinant tRNA_CAG_^Leu ^was expected to decode CUG codons as leucine, higher levels of leucine incorporation were expected at the CUG codon position in the reporter protein. This protein was purified by nickel affinity chromatography and CUG ambiguity was quantified by HPLC-mass spectrometry, as above. Surprisingly, the levels of leucine and serine incorporated in response to the CUG codon in the *PGK *reporter were 28.1% and 71.9%, respectively (Figure 3c,d). Remarkably, however, this dramatic increase in decoding error (28,000-fold) did not significantly decrease growth rate (data not shown).

### Double identity of the CUG codon expands the *C. albicans *proteome

**Figure 4:**
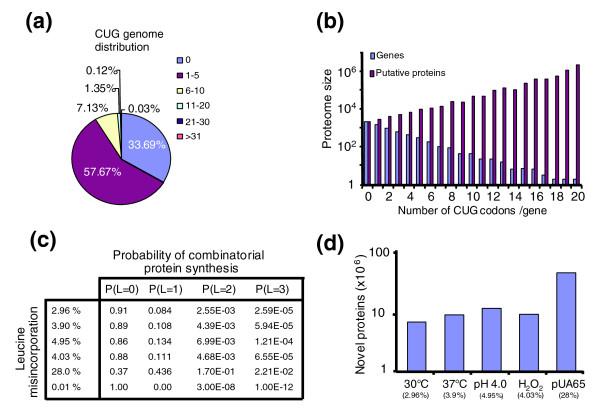
The *Candida albicans *proteome has a statistical nature. **(a) **In *C. albicans*, 33% of the genes do not have CUG codons and 57% have between one and five codons. **(b) **Ambiguous CUG decoding results in exponential expansion of the proteome, allowing the 6,438 *C. albicans *genes to generate 2.8379 × 10^11 ^different proteins. **(c) **The impact of various leucine mis-incorporation levels on the probability of synthesis of proteins with 0, 1, 2, or 3 leucines at CUG positions, for genes containing three CUGs. **(d) **Number of novel proteins generated through ambiguous CUG decoding in the experimental conditions tested. The total number of novel proteins within a cell was estimated as being of 6.7 × 10^6 ^in cells grown at 30°C, of 8.7 × 10^6 ^at 37°C, of 10.9 × 10^6 ^at pH 4.0, of 9.0 × 10^6 ^in the presence of hydrogen peroxide (H_2_O_2_), and of 40 × 10^6 ^in the highly ambiguous cells. 0.01% indicates background decoding error.

The discoveries that *C. albicans *tolerates up to 28.1% of leucine mis-incorporation (Figure 3c,d) and that wild-type cells mis-incorporate leucine at 3% to 5% under standard and mild stress conditions (Figure 3a,b) raised the intriguing issue of proteome complexity in *C. albicans*. In other words, how many different proteins can be generated from the 6,438 *C. albicans *genes? To address this important question, we conducted a detailed survey of the global distribution of CUGs in the *C. albicans *genome. There are 13,074 CUG codons in the haploid genome of *C. albicans*, distributed over 66% of its genes, at a frequency of 1 to 38 CUGs per gene (Figure 4a), with an average of three CUGs per gene. A genome-wide codon-context survey did not identify any particular context bias for the CUG codon (see Additional data file 2), suggesting that leucine and serine are inserted randomly at CUG positions. Therefore, the total number of different proteins that can be generated from ambiguous CUG decoding is 2^*n *^(*n *= total number of CUGs per gene). This implies that the size (diversity) of the *C. albicans *proteome expands exponentially with the number of CUG codons per gene, and that the 6,438 protein-encoding genes of *C. albicans *have the potential to produce a staggering 2.8379 × 10^11 ^different proteins through CUG ambiguity (Figure 4b). In other words, each protein is represented by a mixture (array) of molecules containing leucine or serine at positions encoded by CUG codons. This is of profound biologic significance because it implies that each *C. albicans *cell has a unique combination of proteins.

An important characteristic of the *C. albicans *proteome is that small differences in leucine mis-incorporation have large effects on proteome expansion and diversity. This effect results from the binomial probability of one gene with *n *CUG codons having *i *leucines incorporated at these CUG positions (see Materials and methods, below). To illustrate this, we calculated the probability of synthesis of different proteins for number of leucines 0, 1, 2, and 3; for genes containing three CUGs; and for ambiguity levels of 2.96% (cells grown at 30°C), 3.9% (cells grown at 37°C), 4.95% (cells grown at pH 4.0), 4.03% (cells grown in presence of H_2_O_2_), and 28.1% (pUA65 cells; Figure 4c). Indeed, the probabilities of such a protein to contain one leucine in cells grown at 30°C, 37°C, pH 4.0 and H_2_O_2 _are 8.36%, 10.8%, 13.4% and 11.1%, respectively. In engineered highly ambiguous cells (28.1% leucine mis-incorporation), 43% of the proteins contain at least one leucine at one of the CUG positions (Figure 4c).

**Figure 5:**
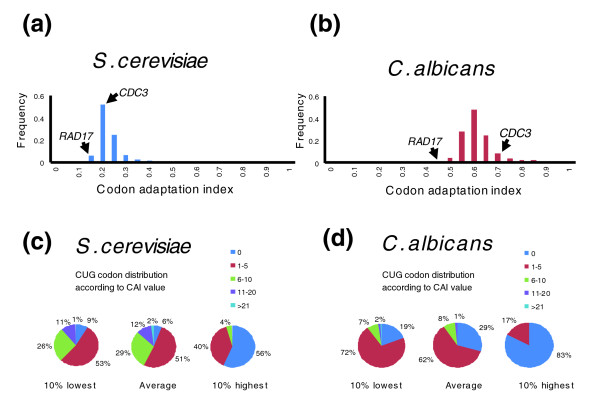
Distribution of CAI values for *Saccharomyces cerevisiae *and *Candida albicans*. The codon adaptation index (CAI) values for the genes of both **(a) ***S. cerevisiae *and **(b) ***C. albicans *genes were determined using the ANACONDA algorithm [66]. The CAI value is a measure of synonymous codon usage bias, which was obtained by extracting the codon usage frequencies from a set of reference genes, and scoring each gene according to its codon usage value [67]. In general, *C. albicans *CAI values were greater than those of *S. cerevisiae*. **(c,d) **The distribution of CUG codons per gene according to their CAI ranking order. In *C. albicans*, CUG codons were strongly underrepresented in the 10% of genes with higher CAI values.

We also calculated the direct impact of ambiguous CUG decoding on expansion of the *C. albicans *proteome by taking advantage of the 'codon adaptation index' (CAI; Figure 5a-d). In *S. cerevisiae*, the 10% of the proteins with the highest CAI values are represented by 50,000 molecules/cell, whereas the 10% of the proteins with the lowest CAI values are represented by 5,000 molecules/cell [24]. Because *S. cerevisiae *and *C. albicans *are close relatives, we used these values as reference for protein expression levels in the latter. For this, the global distribution of CAI values was calculated for *C. albicans *(Figure 5a). In *C. albicans*, CAI values had a broader distribution toward higher values, indicating that its genes often use a small subset of codons to optimize gene expression. We then assumed the following: all *C. albicans *genes are expressed; the abundance of proteins is 5,000 molecules/cell for the 10% of genes with lowest CAI values; the abundance of proteins is 50,000 molecules/cell for the 10% of genes with highest CAI values; and the abundance of proteins is 20,000 molecules/cell for the remaining 80% of genes. This permitted estimation of the number of different protein molecules that could be present within a *C. albicans *cell according to their level of expression. On the basis of CAI distribution for *C. albicans *(Figure 5a,b), we estimated that for CUG mis-translation levels of 2.9% and 28.1% the 6,438 *C. albicans *genes will produce 6 × 10^6 ^and 40 × 10^6 ^proteins, respectively (Figure 4d).

**Figure 6:**
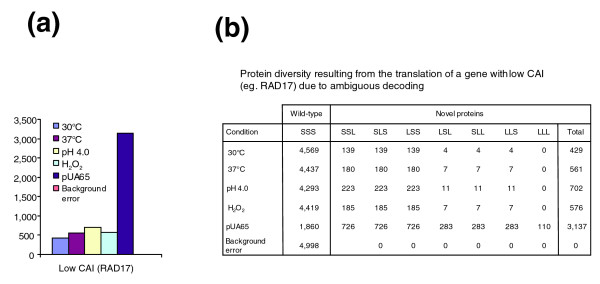
Calculation of the number of novel proteins that can be produced by ambiguous decoding of low CAI mRNAs. **(a) **Novel proteins arising from ambiguous decoding of mRNAs encoded by genes with low codon adaptation index (CAI) value in the different physiologic conditions indicated. The *RAD17 *gene, containing three CUG codons, was used as an example of a gene with a low CAI, because its CAI value falls within the range of values exhibited by the 10% of genes with lowest CAI value in *Candida albicans *(CAI_RAD17 _= 0.448). This set of genes produce approximately 5,000 protein molecules *in vivo *in yeast [24]. **(b) **Total number of different proteins that can be generated from ambiguous CUG decoding. The probability of different proteins that arise from genes containing CUGs, caused by serine or leucine insertion at CUG positions, was calculated as described in the Materials and methods section. In this case, of the 5,000 Ra17p molecules synthesized, 4,569 are wild-type and 429 are novel molecules (8.6%). The data unequivocally show that *C. albicans *proteins are quasi-species [43] and that its proteome has a statistical nature.

**Figure 7:**
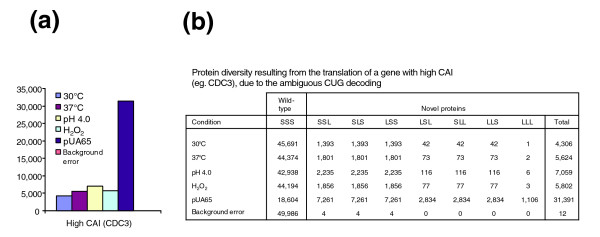
Calculation of the number of novel proteins that can be produced by ambiguous decoding of high CAI mRNAs. **(a) **Number of novel proteins synthesized by ambiguous CUG decoding of genes with high codon adaptation index (CAI) value in the different physiologic conditions indicated. The *CDC3 *gene, which contains three CUG codons, was used as an example of a gene with a high CAI value (CAI_CDC3 _= 0.694) for *Candida albicans*. This set of genes produces approximately 50,000 protein molecules *in vivo *in yeasts [24]. **(b) **Table showing the number of different protein molecules that arise from ambiguous CUG decoding of *CDC3*, following the methodology described in the Materials and methods section. In this case, for 2.9% of CUG ambiguity, of the 50,000 Cdc3p molecules synthesized, 45,691 are wild type whereas 4,306 are novel molecules (8.6%), containing a combination of 1, 2, or 3 leucines at the three CUG positions. The data show that *C. albicans *proteins are quasi-species [43] and that its proteome has a statistical nature.

The proteome analysis was extended one step further to compare the impact of CUG ambiguity in abundant and rare proteins. *CDC3 *and *RAD17* genes, whose CAI values (0.69 and 0.448, respectively) are at the high and low extremes of the distribution of CAI values for *C. albicans *(Figure 5a,b), were chosen for this analysis. Ambiguous CUG decoding had a stronger impact on *CDC3 *than on *RAD17*, indicating that highly expressed proteins encoded by genes with high CAI values are affected the most. Indeed, for 2.9% ambiguity, Rad17p is represented by 4,569 wild-type and 429 novel polypeptides (8.58%), whereas Cdc3p is represented by 45,691 wild-type and 4,306 novel polypeptides (8.6%), containing a combination of one, two, or three leucines at the three CUG positions (Figures 6 and 7). Overall, approximately 10% of the proteins synthesized from mRNAs containing three CUG codons are novel. Interestingly, codon usage analysis showed that CUG codons are highly under-represented in 10% of *C. albicans *genes with the highest CAI values, but are used frequently in 10% of the genes with the lowest CAI values (Figure 5c,d). Furthermore, 83% of *C. albicans *genes with the highest CAI do not have CUG codons, whereas 81% of genes with the lowest CAI have at least one CUG. This is in sharp contrast to CUG usage in *S. cerevisiae*, in which only 56% of genes with highest CAI and 6% of genes with average CAI did not have CUGs.

### Ambiguous CUG decoding generates phenotypic diversity

**Figure 8:**
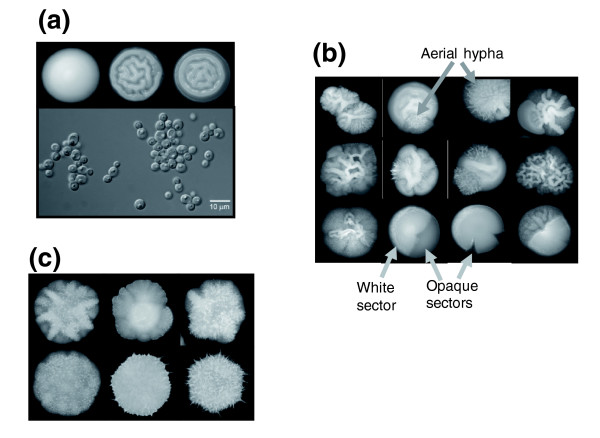
Ambiguous CUG decoding generates phenotypic diversity. **(a) ***Candida albicans *control cells (pUA12) grew in agar plates as white, smooth, or slightly rough colonies. **(b) **Expression of the *Saccharomyces cerevisiae *tRNA^Leu ^(pUA15) in *C. albicans *resulted in 88.9 ± 4.3% morphogenesis (data not shown), with appearance of an array of morphologic phenotypes. Morphology variation was characterized by appearance of large sectors containing opaque cells and aerial hyphae and by formation of unusual morphologic structures in the colonies. **(c) **Colonies with homogeneous morphology isolated from sectors of colonies shown in panel b. In panels a and b, phenotypic variability was determined on agar plates after 7 days of growth, considering all morphologic changes that deviated from the white smooth phenotype, which is characteristic of *C. albicans *wild-type cells.

*C. albicans *cells grow on agar plates as white smooth or slightly wrinkled colonies (Figure 8a). They can acquire alternative morphologies at low frequency (10^-4 ^to 10^-1^) when they are exposed to both physical and chemical agents, namely serum, low pH, nutrient starvation, high temperature, and UV light [25]. These morphologies range from smooth to various wrinkled forms, and result from induction of hypha development inside the colonies. Also, some strains are able to switch from the typical white form to an alternative form termed opaque [26]. Opaque cells are larger, have different gene expression profiles, and are less virulent than white cells. They are also homozygotic for the mating locus (*MTL*; *AA *or *αα*) and are able to mate, while white cells are heterozygotic (*A*/*α*) and do not mate [27].

**Figure 9:**
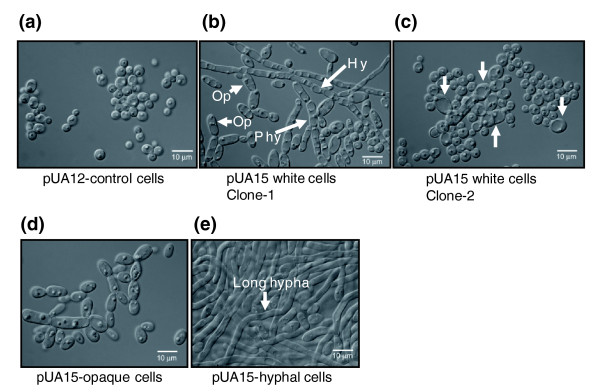
Morphologic diversity of highly ambiguous *Candida albicans *cells in liquid culture. **(a) ***C. albicans *CAI-4 control cells. **(b,c) **Cells transformed with the pUA15 plasmid, carrying a *S. cerevisiae *tRNA_CAG_^Leu^, exhibited diverse morphologic types that ranged from large circular or ovoid opaque-like cells (Op) that contained large vacuoles, to pseudo-hyphal (Phy) and hyphal forms (Hy; arrows). **(d) **Opaque cells (ovoid) isolated from sectors of white colonies maintained in minimal media. **(e) **A small percentage of the pUA15 clones produced very long hypha.

Ambiguous CUG decoding exposed hidden phenotypic diversity without any chemical or physical inducer. Indeed, a high percentage of the colonies of the pUA65 clone, expressing the *S. cerevisiae *leucine CUG decoding tRNA_CAG_^Leu^, but not the cells transformed with plasmid pUA63 (lacking the *S. cerevisiae *tRNA_CAG_^Leu^), exhibited highly variable morphologies characterized by formation of aerial hyphae and white-opaque sectoring (data not shown). To exclude eventual secondary effects caused by the *PGK *reporter gene in the phenotypic variation observed, we have constructed two new plasmids that lack the reporter gene, namely a plasmid containing the *S. cerevisiae *tRNA_CAG_^Leu ^gene only (pUA15) and a control plasmid that does not contain the heterologous tRNA_CAG_^Leu ^gene (Additional data file 3 [Figures S3A,B]). Again, 88% of the colonies of the pUA15 clone, expressing the *S. cerevisiae *leucine tRNA_CAG_^Leu ^gene, exhibited highly variable morphologies characterized by formation of aerial hypha and white-opaque sectoring (Figure 8b,c). Colonies of pUA12 clones (control plasmid) did not show this phenotypic variability and were similar to untransformed CAI-4 cells (Figure 8a). Approximately, 40% of the pUA15 clones produced hypha that penetrated deeply into agar, and 40% to 50% (depending on the clone) produced opaque sectors that frequently occupied 20% or more of the colony. In some colonies the entire surface was covered with long aerial hyphae (Figure 8b) and cells from these colonies formed very long filaments and flocculated when grown in liquid media (data not shown), suggesting that they were highly hydrophobic. Cells from colonies with alternative morphologies also exhibited strong morphologic variability. Each colony was composed by a mixture of yeast-like cells, pseudophyphae, and hyphal cells in various proportions, depending on the clone (Figure 9a-e). Large cells and ovoid-elongated cells were often observed, suggesting that these colonies contained a mixture of opaque and white cells (Figure 9b-e).

Considering that increased CUG ambiguity induced extensive morphologic variation and that *C. albicans *plasmids lack a centromere and are inherently unstable, we tested whether random integration of the pUA15 plasmid in the *C. albicans *genome could be responsible for the phenotypes observed. For this, we selected clones that could rapidly lose the pUA12 or pUA15 plasmids (nonintegrated plasmids) using minimal medium containing uridine plus 5-fluoro-orotic acid (5-FOA) [28]. Because clones that maintained the plasmids (pUA12 or pUA15) would die in presence of 5-FOA as a result of expression of their *URA3 *selective marker gene, we were able to confirm whether plasmid loss would result in disappearance of the phenotypic diversity observed. Indeed, CAI-4 untransformed as well as pUA12 and pUA15 transformed cells that grew in 5-FOA (lost the plasmid) did not exhibit morphologic variation (Additional data file 4 [Figures S4A-D]). To ensure further that the above-mentioned spurious plasmid integrations did not affect phenotypic variability through eventual disruption of one of the copies of the endogenous serine tRNA_CAG_^Ser ^gene, we checked the integrity of this gene by PCR amplification of its locus. No disruption was observed in the clones tested (Additional data file 5 [Figures S5A-C]). Finally, the high level of white-opaque switching prompted us to verify the conformation of the mating locus of our *C. albicans *CAI-4 strain. Because only homozygotic *MTLAA *or *MTLαα *cells can switch from the white to the opaque phenotype [29,30], we checked whether the original strain was *MTL *homozygotic. For this, the *OBPα *and *MTLA1 *genes were amplified by PCR. Untransformed CAI-4 cells or cells transformed with the pUA12 control plasmid were heterozygotic *MTLAα*, but two pUA15 clones tested were homozygotic *MTLαα *(Additional data file 6 [Figures S6A,B]). These findings, plus the inability of the pUA12 plasmid to induce phenotypic variation, confirmed that CUG ambiguity is an authentic generator of phenotypic diversity in *C. albicans*.

We attempted to isolate colonies that could maintain homogeneous morphologies by removing cells from sectors of pUA15 clones and re-plating them on fresh agar (Figure 8c). However, there was always high reversion and switching between different morphologies. This was in accordance with the statistical nature of the *C. albicans *proteome and it is likely that the main role of the dual identity of the tRNA_CAG_^Ser ^is to generate phenotypic diversity. It raises the hypothesis that CUG ambiguity created by this unique tRNA may increase adaptation potential and allow *C. albicans *to escape the immune system by continuously rearranging its surface antigens.

## Discussion

### Implications for the evolution of the genetic code

Genetic code alterations pose unanswered questions about the mechanisms by which they evolve, and their potential selective advantage and physiologic acceptability. We chose the *Candida *genetic code change as a molecular and cellular model to elucidate those questions. This and previous studies [17,31-33] strongly support the hypothesis that genetic code alterations evolved through ambiguous codon decoding mechanisms [16,34].

Ambiguous CUG decoding in *C. albicans*, which results from mis-charging of the tRNA_CAG_^Ser^, proved interesting from a structural perspective, because it is not yet clear how this novel tRNA is recognized by the LeuRS and why this enzyme fails to edit the mischarged leu-tRNA_CAG_^Ser^. Archeal and most eukaryotic LeuRSs recognize the long variable arm of cognate tRNA^Leu ^[35], whereas the yeast LeuRS makes direct contact with the methyl group of m^1^G_37 _and with A_35 _in the anticodon-loop and nonspecific contacts with the phosphate backbone of the anticodon stem [21,36]. Like canonical tRNA^Leu^, tRNA_CAG_^Ser ^contains A_35 _and m^1^G_37 _in its anticodon loop. However, the discriminator base is G_73 _(as in other tRNA^Ser^) and not A_73 _(as in tRNA^Leu^), which should prevent its recognition by the *C. albicans *LeuRS. This is of particular relevance because changing A_73 _to G_73 _in both yeast [36] and human tRNA^Leu ^[37,38] changes its identity from leucine to serine. In the *Pyrococcus horikoshii *LeuRS-tRNA^Leu ^complex, A_73 _is recognized by the amino acid residue 504 of the editing domain and the interaction is disrupted when A_73 _is replaced by G_73 _[35]. It is possible that the *C. albicans *LeuRS evolved a novel mechanism for recognizing both G and A at position 73. Regarding the failure of LeuRS to edit mis-charged leu-tRNA_CAG_^Ser^, the LeuRS binds its cognate amino acid (leucine), activates it (as normal), and transfers it to the tRNA_CAG_^Ser ^(see above). In other words, both leucine and tRNA_CAG_^Ser ^are cognate substrates for the LeuRS and consequently the post-transfer editing mechanism is not activated. This is supported by the high degree of amino acid conservation between LeuRS of *C. albicans *and those of other yeasts, particularly within the editing domain. Functionally, the *S. cerevisiae CDC60 *(LeuRS) gene could also be complemented by its *C. albicans *homolog [39].

### Implications of CUG ambiguity for *C. albicans *biology

*C. albicans *is a diploid polymorphic commensal opportunist that causes infection in immune compromised hosts. Morphologic variation, growth at high temperature, yeast-hypha transition, proteinase and lipase secretion, and various adhesins all play important roles in infection [40-42]. The phenotypic diversity induced by CUG ambiguity was unanticipated, but it is not yet clear whether it is relevant to pathogenesis. To clarify this important new question, novel reporter systems for monitoring CUG ambiguity *in vivo *during infection will have to be developed. Nevertheless, the phenotypic diversity generated by CUG ambiguity also suggests that genetic code ambiguity has a strong impact on *C. albicans *gene expression, which may in part explain the morphologic diversity observed (see below). However, the multiplicity of forms of *C. albicans *pUA15 cells in liquid and agar cultures complicates quantitative analysis of the link between CUG ambiguity and phenotypic diversity because of differences in gene expression between cells present in the same culture. The exponential increase in the size of the *C. albicans *proteome may ultimately be the main factor contributing to morphologic variation (see below). However, one cannot exclude the hypothesis that CUG ambiguity may activate a master regulator or signalling pathway that regulates morphogenesis in *C. albicans*. This should be clarified by stabilizing some of the morphologies (Figure 8b,c) and comparing the gene expression profiles of each morphotype with that of control cells.

The most remarkable consequence of CUG ambiguity is the exponential expansion of the *C. albicans *proteome. This is of profound biologic significance because arrays of proteins are generated from single mRNAs creating a statistical proteome. It implies that *C. albicans *proteins are quasi-species [43] and that the probability of finding two identical cells in a population is extremely small. It also implies that the *C. albicans *proteome is unstable, and it will be most interesting to determine whether such instability affects genome stability because the latter is notoriously unstable in this human pathogen [44,45]. Our data leave no doubt that important proteome diversity can be generated by small increases in CUG decoding ambiguity. We have found slight increases in CUG ambiguity under stress, in particular at low pH (4.95%), suggesting that the relative activity of the LeuRS increases under stress (Figure 3b). At this point it is not clear how this is achieved, but in *S. cerevisiae *the LeuRS is processed by yscY endopeptidase, which cleaves and inactivates it [46]. Also, the two alleles of the *C. albicans CaCDC60 *gene (LeuRS) are under control of divergent promoters (data not shown), suggesting that LeuRS expression and activity may be modulated by transcriptional and post-transcriptional regulatory mechanisms.

### Genetic code ambiguity as a generator of phenotypic diversity

In yeast, codon ambiguity successfully induces the stress response and increases tolerance to high temperature, lethal doses of heavy metals, and drugs [33]. In an earlier described case, inactivation of the heat shock protein (Hsp)90 molecular chaperone in *Drosophila melanogaster *and *Arabidopsis thaliana *allowed expression of polymorphic proteins that are involved in cell signalling pathways and generated phenotypic diversity [47-50]. In *S. cerevisiae *and *C. albicans*, Hsp90 plays a critical role in drug resistance by maintaining mutant drug resistance genes in a functional state [51]. In another example, proteome disruption created by generalized stop codon read-through of genes and pseudogenes, induced by the yeast [*PSI*] prion [52], resulted in morphologic variation and in a combinatorial response to an array of carbon and nitrogen sources and toxic concentrations of metals, salts, and drugs [50,53]. All three cases - Hsp90 inhibition, [*PSI*] prion induction, and genetic code ambiguity - have similar destabilizing impacts on the proteome (they all lead to large scale synthesis/accumulation of aberrant proteins) and increase phenotypic variation. Recent studies showed that mRNA mistranslation in multicellular organisms is associated with disease [54,55]. However, our data clearly indicate that the negative effect of codon ambiguity on the proteome may, under certain physiologic conditions, be overcome by its capacity to generate novel adaptive traits, at least in unicellular organisms.

## Conclusion

Recent reports on the introduction of non-natural amino acids into the genetic code confirm the hypothesis that organisms are highly tolerant to genetic code changes and readily adapt to genetic code ambiguity [32,56-59]. Our study strongly suggests that genetic code ambiguity generates unanticipated proteome expansion and advantageous phenotypes. This supports the hypothesis that earlier expansion of the genetic code, from a small number of amino acids existent in primordial life forms to the 22 encoded by extant organisms, could have been driven by selection through codon ambiguity. This is compatible with the co-evolutionary theory of the genetic code, which postulates that gradual establishment of amino acid biosynthetic pathways permitted gradual incorporation of new amino acids into the code through a mechanism of donation of codons belonging to pre-existing amino acids [13,60]. The statistical proteome and phenotypic changes described herein for *C. albicans *support the hypothesis that gradual codon identity changes will inevitably block lateral gene transfer and create genetic barriers that may result in evolution of new species. This is confirmed by the inability to express heterologous genes in *C. albicans*. If this hypothesis is valid, then the *Candida *genus should have arisen as a direct consequence of this genetic code alteration, thus illustrating how ambiguous expansion of the genetic code could have played a critical role in the evolution of the primordial life forms, whereas general mRNA mistranslation is *de facto *a generator of phenotypic diversity.

## Materials and methods

### Strains and growth conditions

*Escherichia coli *strain JM109 (*recA1 SupE44 endA1 hsdR17 gyrA96 relA1 thi Δ[Lac-proAB] F'[traD36 proAB-lacI lacZ ΔM15*) was used as a host for all DNA manipulations. *C. albicans *CAI-4 (*ura3Δ::imm434/ura3::imm434*) was grown at 30°C in YEPD (2% glucose; 1% yeast extract, and 1% peptone). Transformed *C. albicans *CAI-4 was grown in minimal medium lacking uridine (0.67% yeast nitrogen base without amino acids, 2% glucose, 2% agar and 100 μg/ml of the required amino acids). Growth under suboptimal conditions was performed in MM-uri at 37°C or supplemented with either 50 mmol/l citrate buffer (pH 4.0) or 1.5 mM H_2_O_2 _at 30°C. Opaque cells were grown at 25°C.

### Plasmid construction and transformation

The *C. albicans *plasmids used in this study were based on the stable double ARS pRM1 vector described by Pla and coworkers [61], with the following modifications. A multi-cloning site was inserted (*NruI/EcoRV*) into that plasmid to construct plasmid pUA12. For heterologous expression of the *S. cerevisiae *tRNA_CAG _gene in *C. albicans *CAI-4, a genomic DNA fragment containing the wild-type *S. cerevisiae *tRNA_GAG_^Leu ^gene (90 base pairs [bp]) was cloned into *Apa I*/*Ava III *cloning sites of the pUA12 plasmid. Upstream of this gene, a 250 bp fragment of the 5' flanking *C. albicans *Ser-tRNA_CAG _gene was also inserted at the *XhoI*/*ApaI *cloning sites, yielding the plasmid pUA15. The *S. cerevisiae *tRNA_GAG_^Leu ^gene was then altered by site-directed mutagenesis to change its near cognate anticodon 5'-GAG-3' to the cognate anticodon 5'-CAG-3' for the CUG codon.

The reporter system was constructed on the basis of the *C. albicans CaPGK1 *gene and was assembled into pSL1190 in three cloning steps. First, the promoter and the amino-terminal sequence, encoding the first 69 amino acids of *CaPGK1*, was amplified with the forward primer 5'-ATTAGGAAGCTTAGTGTTGCGTGTGTGTCAG-3' and the reverse primer 5'-TTATCCCTCGAGACCGTTTGGTCTACCCAAG-3', and inserted at the *HindIII *and *XhoI *restriction sites of pSL1190. Second, a cassette containing the CUG codon and the sequence encoding both proteases cleavage sites, along with *XhoI *and *SacII *restriction sites, was inserted into the tail of the forward-primer 5'-ACTAGACCGCGGGATT ATAAAGATGATGATGATAAGAACGACAAATACTCATTAGC-3', which hybridized with *CaPGK1*. The reverse primer 5'-ATTAGATCGCGATTAGTGATGGTGAT GGTGATGGTTTTTGTTGGAAAGAGCAAC-3' had a six-histidine tail to aid protein purification by nickel affinity chromatography. This second fragment was cloned into the pSL1190 plasmid containing the first fragment at the *XhoI *and *NruI *restriction sites. Finally, the 3'-untranslated region sequence of *CaeEF1-α *was amplified with the forward primer 5'-CTCAACTCGCGAGCTAGTTGAATATTATGTAAGATCTG-3' and the reverse primer 5'-AATTTTCTGCAGCCTTTTGGTGTACGAGAG-3', and cloned into the *NruI *and *PstI *restriction sites of the plasmid from above. Once assembled in the pSL1190, the whole reporter protein was subcloned into the *HindIII *and *PstI *restriction sites of both pUA12 and pUA15. This yielded plasmids pUA63 and pUA65, respectively, which were used to determine CUG decoding ambiguity in *C. albicans*. DNA amplifications were carried out using a Mastercycle gradient (Eppendorf) and standard PCR protocols, and all the cloning was done as described by Sambrook and coworkers [62]. Transformation of *E. coli *was carried out as described by Sambrook and coworkers [62], and *C. albicans *CAI-4 transformation was performed by the spheroplast method, as described in the [63].

### Protein purification and digestion

Cells from overnight cultures were collected by centrifugation and lysed in 100 mmol/l NaH_2_PO_4_, 10.0 mmol/l Tris-Cl (pH 8.0), 8.0 mol/l urea, 2.0 mmol/l PMSF and complete mini EDTA-free protease inhibitor cocktail (Roche, Basel, Switzerland), using glass beads and a BeadBeater (Biospec Products, Bartlesville, OK, USA), with 15 cycles of 1 minute beating and 3 minutes resting on ice. The His-tagged reporter protein was purified by Ni-NTA agarose chromatography, as described by the manufacturer (Qiagen, Hilden, Germany). After fractionation on SDS-PAGE, the band corresponding to the reporter protein was cut and in-gel digested, as described by Kussmann and Roepstorff [64], except that the proteases used were enterokinase and thrombin (Novagen-Merck, Darmstadt, Germany) and the cleavage buffer was a 20 mmol/l Tris-Cl (pH 7.6), 0.15 mol/l NaCl, and 2.5 mmol/l CaCl_2 _solution.

### Mass spectrometry and data analysis

Mass spectra were collected using a Micromass Q-ToF Micro (Waters, Milford, MA, USA) equipped with a nanoeletrospray ion source coupled to a nanoflow HPLC system (CapLC; Micromass). Synthetic peptides with amino acid sequences identical to that of the CUG-reporter peptide were used as mass fingerprint controls in all experiments. The identity of the peptides was determined by tandem mass spectrometry analysis. The spectra were analyzed with Masslynx software version 4.0 from Micromass. Peaks corresponding to leucine and serine containing peptides of +3 and +2 charges with m/Z of 508.56, 762.35, 499.88 and 749.32, respectively, were analyzed. The percentage of leucine incorporation at the CUG codon position was calculated as the fraction of the leucine peptide present in the mixture of both leucine and serine peptides. Three or four independent measurements were taken for quantification of leucine and serine incorporation at the CUG codon positions. An analysis of variance (ANOVA) of the data obtained was performed; when the null hypothesis of equal variances within groups of the ANOVA was rejected, the *post-hoc *Scheffe's test was used and the *P *values determined. In order to ensure that only the CUG codon was misread, the peaks corresponding to hypothetical peptides resulting from misreading of cognate codons by near-cognate tRNAs, namely of the aspartate-GAU codon as glutamate and the lysine-AAA codon as asparagine, were screened in the mass spectrum.

### Bioinformatics analysis of the genome and proteome

The *C. albicans *genome (assembly 19; haploid version), containing 6,438 annotated ORFs, was downloaded from the *Candida *Genome Database [65] and analyzed with ANACONDA [66]. This in-house built software package counted all codons present in the annotated ORFs. The probability of different proteins being generated from genes containing CUGs because of serine or leucine insertion at those CUG positions was calculated using the binomial distribution (b_(*i*,*n*,*P*)_):

$${\text{B}}_{\left(i,n,P\right)}=\frac{n!}{i!\left(n-1\right)!}P^{i}{\left(1-P\right)}^{n-i}$$

Where *n *is the total number of CUG codons per gene, *P *is the probability of leucine incorporation at CUG positions for different percentages of ambiguity, and *i *is the number of CUGs decoded as leucine. (For example, for genes containing three CUGs, *n *= 3 and *i *= 0, 1, 2, or 3.) The total number of novel proteins in the proteome of *C. albicans *was estimated taking into consideration the studies of Ghaemmaghami and colleagues [24], who calculated the correlation between protein abundance and CAI and showed that protein abundance in yeast ranges from 50 up to more than 10^6 ^molecules per cell. We have assumed the following: all *C. albicans *genes are expressed; the abundance of proteins (*N*_*total*_) is 5,000 molecules/cell for the 10% of genes with the lowest CAI values; the *N*_*total *_is 50,000 molecules/cell for the 10% of genes with the highest CAI values [24]; and the *N*_*total *_is 20,000 molecules/cell for the remaining 80% of genes. The number of novel proteins arising (*N*_*novel*_) for each gene was given by the following equation:

*N*_*novel *_= *N*_*total *_× (1 - *b*_(0,*n*,*P*)_); where b_(0,*n*,*P*) _is the the probability of polypeptides having no leucine at CUG codons.

### Phenotypic diversity analysis

*C. albicans *cells grown overnight at 30°C in MM-uri were serially diluted to 1,000 cells/ml. Approximately 50 cells were plated onto fresh agar plates and then allowed to grow at 30°C for 7 days in a humidified incubator to prevent drying of the agar surface. Sectored colonies exhibiting atypical morphology were scored and the data were analyzed for significance using ANOVA. Colonies were photographed using a Stemi 2000-C dissecting microscope equipped with AxioVision Software and a AxioCam HRc camera from Zeiss (Munich, Germany). Cells were photographed using a Zeiss MC80 Axioplan2 light microscope.

## Abbreviations

ANOVA, analysis of variance; bp, base pairs; CAI, codon adaptation index; EF, elongation factor; 5-FOA, 5-fluoro-orotic acid; H_2_O_2_, hydrogen peroxide; HPLC, high-pressure liquid chromatography; Hsp, heat shock protein; LeuRS, leucyl-tRNA synthetase; ORF, open reading frame; PCR, polymerase chain reaction; Sec, selenocysteine; Sep, O-phosphoseryl.

## Authors' contributions

ACG, IM, and GRM carried out experimental work. RMS and GRM contributed to data discussion. AK and BT helped with mass spectrometry analysis. MASS wrote the manuscript, supervised the study, and contributed to the experimental design.

## Additional data files

The following additional data are available with the online version of this paper. Additional data file 1 is a figure showing maps of the pUA63 and pUA65 plasmids that were used to quantify CUG decoding ambiguity in *C. albicans*. Additional data file 2 is a figure of CUG codon context in various yeast species, including *C. albicans*. Additional data file 3 is a figure of the maps of pUA12 and pUA15 plasmids that were used throughout the study. Additional data file 4 is a figure showing that elimination of the pUA15 vector in 5-FOA selective media results in disappearance of phenotypic diversity. Additional data file 5 is a figure showing that the pUA15 plasmid did not alter the tRNA_CAG_^Ser ^locus. Additional data file 6 is a figure of the amplification of the *MTL *locus of CAI-4/pUA12 and CAI-4/pUA15 cells.

## Supplementary Material

Additional data file 1 Presented is a figure showing maps of the pUA63 and pUA65 plasmids that were used to quantify CUG decoding ambiguity in *C. albicans* Click here for file

Additional data file 2 Presented is a figure of CUG codon context in various yeast species, including *C. albicans*. Click here for file

Additional data file 3 Presented is a figure of the maps of pUA12 and pUA15 plasmids that were used throughout the study. Click here for file

Additional data file 4 Presented is a figure showing that elimination of the pUA15 vector in 5-FOA selective media results in disappearance of phenotypic diversity. Click here for file

Additional data file 5 Presented is a figure showing that the pUA15 plasmid did not alter the tRNA_CAG_^Ser ^locus. Click here for file

Additional data file 6 Presented is a figure of the amplification of the *MTL *locus of CAI-4/pUA12 and CAI-4/pUA15 cells. Click here for file
