# Supplementary material for: A genetic code alteration generates a proteome of high diversity in the human pathogen Candida albicans
Source: Genome Biol. 2007 Oct 4;8(10):R206. doi: 10.1186/gb-2007-8-10-r206 (PMC2246281; doi:10.1186/gb-2007-8-10-r206)
Supplement: Additional data file 1 — Presented is a figure showing maps of the pUA63 and pUA65 plasmids that were used to quantify CUG decoding ambiguity in C. albicans [file gb-2007-8-10-r206-S1.doc]

**Figure S1. Map of the pUA63 and pUA65 plasmids.** In order to quantify CUG decoding ambiguity in *C. albicans* a CUG-reporter protein, based on the C. *albicans* *PGK1* gene was constructed, as described in materials and methods. It was sub-cloned in the Hind*III* and Pst*I* restriction sites of the plasmids pUA12 and pUA15, yielding the pUA63 and the pUA65, respectively (see Additional file 3). These plasmids, which were used for expression of the recombinant reporter protein in *C. albicans*, are based on the *C. albicans* pRM1 shuttle vector.
