# Supplementary material for: A genetic code alteration generates a proteome of high diversity in the human pathogen Candida albicans
Source: Genome Biol. 2007 Oct 4;8(10):R206. doi: 10.1186/gb-2007-8-10-r206 (PMC2246281; doi:10.1186/gb-2007-8-10-r206)
Supplement: Additional data file 2 — Presented is a figure of CUG codon context in various yeast species, including C. albicans. [file gb-2007-8-10-r206-S2.doc]

**Figure S2. The context of the *C. albicans* CUG codon is similar to that of other fungal species**. The context of CUG codons of *S. cerevisiae*, *S. pombe, A. fumigatus, C. albicans, S. bayanus, S. mikatae, S. paradoxus, C. glabrata, D. hansenii* and *K. lactis* was analysed using our Anaconda Software built in house. The colour coded map shows that the context of the CUG codon in *C. albicans* and in other fungal species is very similar, as the positive (green) and negative (red) neighbour preferences have similar patterns. The minor differences shown were also present in other species.
