# Supplementary material for: A genetic code alteration generates a proteome of high diversity in the human pathogen Candida albicans
Source: Genome Biol. 2007 Oct 4;8(10):R206. doi: 10.1186/gb-2007-8-10-r206 (PMC2246281; doi:10.1186/gb-2007-8-10-r206)
Supplement: Additional data file 3 — Presented is a figure of the maps of pUA12 and pUA15 plasmids that were used throughout the study. [file gb-2007-8-10-r206-S3.doc]

**Figure S3. Maps of the pUA12 and pUA15 plasmids, A and B)** The pUA12 plasmid was generated by inserting a multi-cloning site (Nru*I*/EcoR*V*) into the pRM1 plasmid [1]. For this, the promoter region of the *CaLEU2* gene was removed with the restriction enzymes Nru*I* and EcoR*V* and a multi-cloning site, with the sequence 5’-GCTAGCGGCCGCTCGAGCTCCGCGGGCCCGGCCGATATCTAGAT CTATGCA T-3’, was inserted into the plasmid, by ligation of a complementary pair of primers. This multi-cloning site had restriction sites for the Nhe*I*, Not*I*, Xho*I*, Sac*I*, Sac*II*, Apa*I*, Xma*I*, EcoR*V*, Xba*I*, Bgl*II* and Ava*III* enzymes. For heterologous expression of the *S. cerevisiae* tRNA genes in *C. albicans* CAI-4, a genomic DNA fragment containing the *S. cerevisiae* tRNA_GAG_^Leu^ gene (90 bp) was amplified by PCR using *S. cerevisiae* genomic DNA as template with the forward primers 5’-GCTATGGGCCCT AGTTG CAACGGTACTCTGGCCGAGTGGTCTAAGGCGTCAGGTTCAGGTCC-3’ and the reverse primer 5’-ATGCATAAAAACAAAATTTGTT GAAA-3’. The anticodon of the *S. cerevisiae* (5´-GAG-3´) was changed to (5´-CAG-3´) by site directed mutagenesis. Upstream of this gene, a 250 bp fragment of the 5’ flanking *C. albicans* Ser-trna_cag_ gene was inserted. This fragment was amplified by PCR from a *XhoI/ApaI* genomic DNA fragment, with the following primers: 5’-CCGCTCGAGCGGGTATGCA ATCGTTGTCTGTAATGTA-3’ and 5’-GCTATGGGCCCAAGCACAAATGGTTAT G ACAATTG ATG-3’.
