# Supplementary material for: A genetic code alteration generates a proteome of high diversity in the human pathogen Candida albicans
Source: Genome Biol. 2007 Oct 4;8(10):R206. doi: 10.1186/gb-2007-8-10-r206 (PMC2246281; doi:10.1186/gb-2007-8-10-r206)
Supplement: Additional data file 4 — Presented is a figure showing that elimination of the pUA15 vector in 5-FOA selective media results in disappearance of phenotypic diversity. [file gb-2007-8-10-r206-S4.doc]

**Figure S4. Loss of the pUA15 vector eliminates phenotypic diversity.** In order to exclude eventual artefacts caused by ectopic integration of the pUA15 plasmid into the *C. albicans* genome we tested whether plasmid loss would also result in elimination of phenotypic diversity. For this, transformed *C. albicans* cells, both CAI-4/pUA12 and CAI-4/pUA15, were grown in liquid YEPD at 30ºC. After 4 successive transfers into fresh YEPD (media without selection), cells were platted in both YEPD agar plates and in Minimal Medium agar plates, supplemented with amino acids, uridine (uri) and 5-Fluoro-orotic Acid (5-FOA). Colonies were allowed to grow for 5 days at 30ºC. **A, B)** Untransformed and pUA12 transformed *C. albicans* CAI-4 cells formed smooth and slightly wrinkled colonies in YEPD and MM + Uri + 5-FOA solid media, respectively. **C)** *C. albicans* CAI-4 cells transformed with the pUA15 plasmid containing the *S. cerevisiae* tRNA_CAG_^Leu^, which decodes CUG codons as leucine, showed similar phenotypes to those of CAI-4 and CAI-4/pUA12 cells, indicating that plasmid loss results in elimination of the phenotypes described in Figure-8. **D)** The cellular morphology of CAI4-pUA12 grown in MM-uri (containing the plasmid) or MM+uri+5-FOA (without the plasmid) was identical. However, *C. albicans* CAI-4/pUA15 cells formed long hypha in MM-Uri (plasmid present) similar to those described in Figure 9, but had yeast like morphology when grown in MM+Uri+5-FOA (without the plasmid). Therefore, the phenotypic diversity displayed by *C. albicans* cells transformed with the pUA15 plasmid results directly from increased CUG ambiguity and not from random plasmid integration into the *C. albicans* genome. This is in line with the observation that pUA12 transformed cells did not generate phenotypic diversity above background levels.
