# Supplementary material for: A genetic code alteration generates a proteome of high diversity in the human pathogen Candida albicans
Source: Genome Biol. 2007 Oct 4;8(10):R206. doi: 10.1186/gb-2007-8-10-r206 (PMC2246281; doi:10.1186/gb-2007-8-10-r206)
Supplement: Additional data file 5 — Presented is a figure showing that the pUA15 plasmid did not alter the tRNACAGSer locus. [file gb-2007-8-10-r206-S5.doc]

**Supplementary Figure 5. Transformation of *C. albicans* cells with pUA15 plasmid did not alter the tRNA_CAG_^Ser^ locus.** To ensure that the phenotypic diversity observed was not caused by insertion of the plasmid used (pUA15) into the tRNA_CAG_^Ser^ locus, which would disrupt expression of wild type tRNA_CAG_^Ser^, we have amplified this locus from pUA15 transformed clones. The tRNA_CAG_^Ser^ gene is located on chromosome 5, Ca21chr5, positions 66,383 to 66,464, and its sequence and the sequences of upstream and downstream flanking regions are identical in both alleles (*C. albicans* is a diploid fungus). **A)** Two primers were designed to amplify this region, upstream and downstream of the tRNA_CAG_^Ser^ gene, namely CAG_F (5’-TAACAACTAAGCACAGATG-3’) and CAG_R (5’-TTGAATAGTCTGTCCTCTG-3’). **B)** Detailed diagram of the pUA15 construct showing the tRNA gene region containing an upstream DNA fragment that corresponds to the 5’ flanking region of the *C. albicans* tRNA_CAG_^Ser^. This fragment was inserted to ensure efficient transcription of the tRNA gene by DNA polymerase III. This upstream fragment was amplified from the contig19-10080, p66542-66764. The *S. cerevisiae* tRNA_CAG_^Leu^ was amplified from *S. cerevisiae* genomic DNA, it is present in DNA chromosome VII, p700669-700760. **C)** The amplified DNA was run on a labchip gel and the size of amplified bands was identical to that expected from sequence analysis of the chromosome locus. This showed that the *C. albicans* tRNA_CAG_^Ser^ locus was not disrupted by spurious insertion of the vector into the genome. CAI-4/pUA15 and CAI-4/pUA12 cells were analysed. DNA from plasmids pUA15 and pUA12 was used as negative controls for the DNA amplification. The amplified DNA was sequenced to confirm the authenticity of the DNA sequences.
