# Supplementary material for: A genetic code alteration generates a proteome of high diversity in the human pathogen Candida albicans
Source: Genome Biol. 2007 Oct 4;8(10):R206. doi: 10.1186/gb-2007-8-10-r206 (PMC2246281; doi:10.1186/gb-2007-8-10-r206)
Supplement: Additional data file 6 — Presented is a figure of the amplification of the MTL locus of CAI-4/pUA12 and CAI-4/pUA15 cells. [file gb-2007-8-10-r206-S6.doc]

**Figure S6. Amplification of the *MTL* locus of CAI-4/pUA12 and CAI-4/pUA15 cells. A)** The *C. albicans* strain CAI-4 used in this study, transformed with the control plasmid (pUA12), was heterozygous for the *MTL* locus. The left panel shows amplification of the *MTLα* locus and the right panel shows amplification of the *MTLA* locus. As expected, this result indicated that the strain used in our studies was an authentic CAI-4 strain (*MTLA/α*). **B)** The pUA15 clones tested were homozygotic for *MTLAA*, indicating that CUG ambiguity did induce *MTL* homozygoty in *C. albicans*, which is in line with the observation that increased CUG ambiguity induces white-opaque switching.
